# Prevalence of eimeriosis in the one-humped camels (*Camelus dromedarius*) from Riyadh and Al-Qassim, Saudi Arabia

**DOI:** 10.7717/peerj.10347

**Published:** 2020-11-18

**Authors:** Dina M. Metwally, Tahani T. Al-Otaibi, Shurug A. Albasyouni, Manal F. El-Khadragy, Reem A. Alajmi

**Affiliations:** 1 Department of Zoology, College of Science, King Saud University, Riyadh, KSA; 2 Department of Parasitology, Faculty of Veterinary Medicine, Zagazig University, Zagazig, Egypt; 3 Department of Biology, Al-Nairiyah University College, Hafr Al-Batin University, Hafr Al-Batin, Saudi Arabia; 4 Department of Biology, Faculty of Science, Princess Nourah Bint Abdelrahman University, Riyadh, Saudi Arabia; 5 Department of Zoology and Entomology, Faculty of Science, Helwan University, Helwan, Egypt

**Keywords:** *Eimeria* spp., *Camelus dromedarius*, Saudi Arabia

## Abstract

**Background:**

The one-humped camels are economically important for several countries in Africa, Asia, and the Arabian Peninsula. Coccidiosis causes significant economic impact. Studies on coccidian parasite species causing such infections are limited. The present study aimed to carry out a survey of *Eimeria* spp. in camels from Riyadh and Al-Qassim, Saudi Arabia*.*

**Methods:**

A total of 209 fecal samples from *Camelus* (*C.*)* dromedarius* slaughtered in West Abattoir in Riyadh and Onaizah Modern abattoir in Al-Qassim were collected. Samples were examined by flotation methods and oocyst sporulation.

**Results:**

Of the 209 examined fecal samples, 75 were positive for *Eimeria* spp..The prevalence of oocysts in Riyadh and Al-Qassim were 33.89% (40/118) and 38.46% (35/92), respectively. The prevalence in young male camels was 41.02% (32/78) and 39.62% (21/53), respectively and in adult males was 19.35% (6/31) and 36% (9/25), respectively. Adult females displayed a prevalence of 22.22% (2/9) and 38.46% (5/13) in Riyadh and Al-Qassim, respectively. Three *Eimeria* spp. were identified; *E. cameli*, *E. rajasthani*, and *E. pellerdyi*. The presence of *E. pellerdyi* is considered the first record in Saudi Arabia.

## Introduction

Camels are an important source of milk and meat in many counties of the world, mostly in Asia and Africa (FAOSTAT, 2019). The dromedary camel (one-humped camels) comprises approximately 95% of the entire Old-World camel population. These animals are distributed in 47 countries (FAOSTAT, 2019) and play a significant role in the economies of these countries.

Saudi Arabia has experienced a recent substantial growth in the camel population (Faye, 2015), having a population of around 500,000 in 2017, with the most expanding rate observed in the Riyadh region (FAOSTAT, 2019). The dromedary camel plays an important role in the economy, particularly in the culture of Arabian countries. Apart from being adapted to the harsh environment, these pseudo-ruminants, popularly known as “ship of the deserts” are multipurpose animals and utilized for milk and meat production, hair/felt, racing, transportation, and tourism (Faye, 2014; Faye, 2015). Camel production is severely affected by several diseases, particularly in the absence of adequate veterinary services (Megersa, 2010).

*Eimeria* spp. are intracellular protozoan parasites mainly colonizing the gastrointestinal tract and causing diarrhea, weakness, dehydration, and weight loss. Infection may lead to death of camel calves (Hussein, Kasim & Shawa, 1987; Barth, 2003; Al-Megrin, 2015; Al-Afaleq et al., 2018; Abbas et al., 2019).

*Eimeria* spp*.* are monoxenous, requiring only one host to complete their life cycle. The life cycle comprises an exogenous phase (sporogony), involving a free-living phase outside the host, and a parasitic endogenous phase occuring inside the host. In the host, both asexual and sexual reproduction cycles are observed (Chartier & Paraud, 2012). Non-sporulated oocysts pass with the feces. Oocysts sporulate after 2–7 days, according to coccidian species and the environmental conditions; oxygen, temperature, and moisture are the most important factors influencing sporulation (Chartier & Paraud, 2012). Five *Eimeria* spp. are known to infect the camel intestine (Gerlach, 2008; Borji et al., 2009; Djerbouh et al., 2018). Some of these species are widely disseminated, with high prevalence rates among camels (Luckins, 1992; Dubey & Schuster, 2018). The most widespread species of camelid *Eimeria* include *E. dromedarii* and *E. cameli*, while others (*E. pellerdyi, E. rajasthani*, *and E. bactriani*) are present only in selected regions. Species related to disease mainly include *E. cameli* and *E. dromedarii* (Djerbouh et al., 2018). Variation in the distribution of *Eimeria* spp. is influenced by factors such as environmental conditions, animal physiology and health, farming practices, sickness and stress (Barth, 2003). Disease caused by these parasitic species is of vast economic importance for camel husbandry due to enteritis, diarrhea and poor weight gain (Wernery, Kinne & Schuster, 2014).

Traditionally, the morphology of sporulated oocysts has been used for identification of *Eimeria* spp. (Dubey & Schuster, 2018). The criteria for species identification of the oocyst include the size, the shape, and the presence of characteristic elements (polar cap, micropyle, color, aspect of the oocyst wall, oocystal and sporocystal residues, etc.) (Gerlach, 2008).

The distribution of *Eimeria* spp. in camels in Saudi Arabia has been studied to a limited extent, and insufficient data exist regarding species causing infections and inflicting economic losses. The present study focuses on determining the relevance of *Eimeria* spp. infection in the one-humped camels slaughtered at abattoirs in Saudi Arabia.

## Material and Methods

### Ethical statement

The current study was approved by the Institutional Committee of Post-graduate Studies and Research at King Saud University (Saudi Arabia), (IRB number: KSU-SE-18-33).

### Sample collection

Fresh fecal samples were collected between February and October 2018 by veterinarians during post-mortem inspections of slaughtered animals at the West Abattoir in Riyadh and Onaizah Modern Slaughterhouse in Al-Qassim, Saudi Arabia. Fresh formed fecal samples (the amount of feces varied from camel to camel, depending on availability) were isolated from 209 camels (118 from Riyadh and 91 from Al-Qassim) of different age and sex groups and transported to the laboratory in boxes containing ice packs.

### Coprological examination

Fecal samples (3 g from each sample) were examined by direct smear and zinc sulfate flotation (33 g zinc sulfate dry crystals + 67 mL distilled water) as described elsewhere (Dubey & Pande, 1964; Truant et al., 1981). Briefly, the Fresh fecal samples were mixed with tap water, the mixture was subjected to centrifugation (800 × g for 2 min), supernatant was discarded, and the sediment was mixed with zinc sulphate for flotation technique to demonstrate the presence of oocysts.

### *Eimeria* spp. oocysts sporulation

Fresh feces in which *Eimeria* oocysts were identified was mixed with 2.5% aqueous potassium dichromate (K_2_Cr_2_O_7_) at a ratio of one volume of feces to five volumes K_2_Cr_2_O _7_solution. The fecal-dichromate mixture was kept in a Petri dish and maintained at 28 °C for 6–10 days to allow oocyst sporulation. Oocysts were examined daily to follow the sporulation process. After sporulation, 10 mL of the fecal-dichromate mixture was pipetted into a 15-mL conical vial and centrifugated at 1,000× g for 5 min. The pellets were washed with 10 mL of water five times to remove the K_2_Cr_2_O_7_, with centrifugation at 1,000× g for 5 min between washings. Ten milliliters of zinc sulfate flotation solution was then added, and the solution was mixed by vortexing. After centrifuging at 500 × g for 10 min, the top 3 mL of the solution was removed, placed into 50 mL conical centrifuge tubes containing 45 mL of water, and centrifuged at 1,000× g for 10 min to pellet the oocysts. Sporulated oocysts were then resuspended in 0.5 mL of water and stored at 4 °C until further use (Fritzler et al., 2011).

### Statistical analysis

Statistical analysis were performed using the Statistical Package for Social Sciences (SPSS) software (version 17, SPSS, Inc., Chicago, IL, USA). All data were analyzed as a completely randomized design using independent sample *t*-test to compare between data of two experimental groups and One-Way Analysis of Variance (ANOVA) for data of other experimental groups, followed by Duncan’s test to compare the significance between means. Comparisons between means were considered significant at *p* ≤ 0.05. Results were expressed as a mean ± standard error of mean (SEM).

## Results

### Sample collection

A total of 209 slaughtered camels from Riyadh and Al-Qassim regions were examined for the presence of *Eimeria* spp. We did not observe any significant differences between Riyadh and Al-Qassim regions in terms of infection rates. The prevalence of *Eimeria* spp. by study area is displayed in Table 1.

**Table 1:** Prevalence of *Eimeria* spp. in camels from Riyadh and Al-Qassim regions according to age and sex of the camels.

Categories	Regions			
	**Riyadh**		**Al-Qassim**	
	**No.examined**	**No.infected** **(%)**	**No.examined**	**No.infected** **(%)**
Young male camels	78	0.41 ± 0.06^a^ 32 (41.02%)	53	0.40 ± 0.07^a^ 21 (39.6%)
Adult male camels	31	0.19 ± 0.07^a^ 6 (19.3%)	25	0.36 ± 0.10^a^ 9 (36.0%)
Young female camels	NA	NA	NA	NA
Adult female camels	9	0.22 ± 0.15^a^ 2 (22.2%)	13	0.38 ± 0.14^a^ 5 (38.5%)
Total No.	118	0.34 ± 0.04^a^ 40 (33.9%)	91	0.38 ± 0.05^a^ 35 (38.5%)

**Notes.**

NA, not available. Mean values with superscript (a, b) in the same column differ significantly at *p* ≤ 0.05. Comparisons between the same age is between two different gender. Comparisons between the same gender is between two different ages.

The prevalence of parasites in young male camels was 41.02% and 39.6% and 19.3% and 36% in adult males for Riyadh and Al-Qassim, respectively. The infection rate in adult females in the two areas was 22.2% and 38.5%, respectively (Table 1).

Three *Eimeria* spp. were observed among the 75 infected camels; *E. cameli*, *E. rajasthani*, and *E.pellerdyi*. A significant difference was found between the infection rate of *E. rajasthani* and the other two species. In both Riyadh and Al-Qassim, *E. rajasthani* showed the highest infection rate (52% ±8% and 51% ±9% of positive samples, respectively) (Table 2).

**Table 2:** Prevalence of different *Eimeria* spp. in Riyadh and Al-Qassim regions.

**Regions**	**No. of infected camels**	***E. cameli*** **No. of infection (%)**	***E. rajasthani*** **No. of infections (%)**	***E. pellerdyi*** **No. of infections (%)**
**Riyadh**	40	0.20 ±0.06^b^ 8 (20.0%)	0.52 ±0.08^a^ 21 (52.5%)	0.28 ±0.07^b^ 11 (27.5%)
**Al-Qassim**	35	0.29 ±0.08^b^ 10 (28.6%)	0.51 ±0.09^a^ 18 (51.4%)	0.20 ±0.07^b^ 7 (20.0%)

**Notes.**

Mean values with superscripts (a, b) in the same row differ significantly at *p* ≤ 0.05.

### Morphological characteristics of *Eimeria* spp.

#### Eimeria cameli (E. cameli)

**Figure 1:**
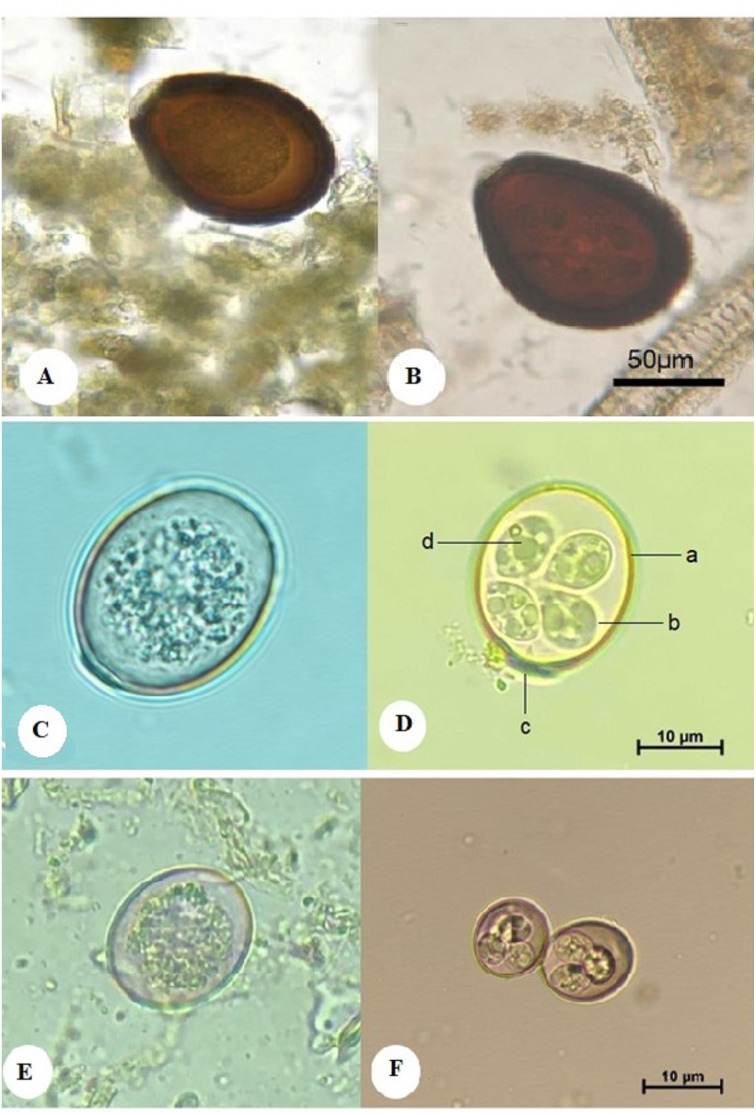
Morphological characteristics of isolated *Eimeria* spp. showing: *E. cameli* oocysts (Bar = 50 µm). (A) Non-sporulated oocyst. (B) Sporulated oocyst, *E. rajasthani* oocysts (Bar = 10 µm). (C) Non-sporulated oocyst. (D) Sporulated oocyst : a. double wall; b. sporocyst; c. micropyle covered with a dome-shaped polar cap; d. sporozoite, and *E. pellerdyi* oocysts (Bar = 10 µm). (E) Non-sporulated oocyst. (F) Sporulated oocyst.

The oocyst of *E. cameli* was large and pear-shaped, measuring 92–112 µm in length and 68–92 µm in width (mean, 102 × 80 µm). The oocyst wall is dark brown and consists of a thin outer and thick inner layer. The micropyle was 10–27 µm wide, displaying no polar granules nor oocystic residual bodies, and it had no polar cap (Fig. 1A). The sporulated oocyst contained four poorly differentiated sporocysts, each measuring 29–33 in length and 20–23 µm in width (mean, 31 × 21.5 µm). Each sporocyst contained two rounded sporozoits, each 8.9–10.3 µm (mean, 9.6 µm) and with sporocystic residual bodies. Sporulation time was six days at 28 °C (Fig. 1B).

#### Eimeria rajasthani (E. rajasthani)

The oocyst was oval, measuring 25–30 in length and 21–24 µm in width. It had a double-layered wall. The micropyle was present and covered with a dome-shaped polar cap (Fig. 1C). The sporulated oocyst contained 4 ellipsoidal sporocysts, each measuring 12–14 in length and 9–10 µm in width (mean, 13 × 9.5 µm), and each contained 2 ovoid sporozoites, each measuring 5–6.6 µm in length and 3–4 µm in breadth (mean, 5.8 × 3.5 µm). Oocystic and sporocystic residual bodies were absent, as were polar granules. The sporulation time was four days at 28 °C (Fig. 1D).

#### Eimeria pellerdyi (E. *pellerdyi )*

The oocyst was ovoid, measuring 20–24 in length and 16–20 µm in width, and it was surrounded by a double wall. The outer wall was reddish brown, while the inner layer was dark green. No micropyle or polar cap was observed (Fig. 1E). The sporulated oocyst had four subspherical sporocysts, each measuring 8.2–8.8 µm in length and 7–7.8 µm in width (mean, 8.5 × 7.4 µm). Each sporocyst had two sporozoites, measuring 4.5–5.5 µm in length and 2.5–3.5 µm in width (mean, 5 × 3 µm). No oocystic residual body was observed. The sporulation time was four days at 28 °C (Fig. 1F). The presence of *E. pellerdyi* is taken into account the first record in Saudi Arabia.

## Discussion

Eimerian parasites are the predominant intestinal tract pathogens in many animals; they invade and damage the intestinal epithelium, causing severe injury and economic losses (Mehlhorn, 2014).

To determine the prevalence and distribution of *Eimeria* spp., the present study examined the fecal samples from 209 camels. Out of the 209 examined fecal samples, 75 were positive for *Eimeria* spp*.*, and so the overall prevalence for the *Eimeria* spp*.* was 35.9%. Three types of *Eimeria* spp*.* were distinguished; *E. cameli* was the largest species of *Eimeria* detected and was characterized by a dark brown, double thick wall and the presence of a micropyle. The other two *Eimeria* spp. were smaller in size; *E. rajasthani* was oval in shape and had a micropyle and a polar cap, whereas *E. pellerdyi* was oval in shape and had neither micropyle nor polar cap. Dependent on morphological characteristics only, no novel *Eimeria* spp. were found. All the isolated oocysts were morphologically similar to those previously identified in *C. dromedarius* (Yagoub, 1989; Gerlach, 2008; Dubey & Schuster, 2018).

The prevalence rate was 33.9% in Riyadh and 38.5% in Al-Qassim. *E. cameli* had a prevalence of 20% in Riyadh and 28.6% in Al-Qassim; *E. rajasthani* had a prevalence of 52.5% in Riyadh and 51.4%in Al-Qassim, and *E. pellerdyi* had a prevalence of 27.5% in Riyadh and 20% in Al-Qassim. Regarding previous studies on *Eimeria* spp. in Saudi Arabia, Kawasmeh & Elbihari (1983) found *E. cameli* oocysts in 146 of 960 fecal samples (prevalence, 14%). Samples were collected twice weekly from an unspecified number of camels for 12 consecutive months. Studying 500 samples from 6 months to 5 years-old camels in four different regions, Kasim, Hussein & Shawa (1985) reported a prevalence of 41.6%, including *E*. *dromedarii*, (28.4%) and *E*. *rajasthani* (22.2%). Hussein, Kasim & Shawa (1987) examined 385 samples identifying a prevalence of 40%; *E*. *dromedarii* was the most prevalent, followed by *E*. *rajasthani*, and *E*. *cameli*; however, the relative figures were not stated. Clinical signs were observed in young camels. Mahmoud et al. (1998) examined 240 samples, observing that 15.7% of 83 adults and 10.2% of calves were infected. In adult camel*s, E. cameli* was found in 2.4%, *E. rajasthani* in 7.2%, and *E. dromedarii* in 12%. In calves, *E. cameli,* was found in 1.3%, *E. rajasthani* in 5.1%, and *E*. *dromedarii* in 6.3%.

The climate of Riyadh is known for its temperature extremes, with low humidity during the year, especially in the summer season. The temperature varies significantly between night and day: In the summer, the highest average temperature ranges between 40 °C and 43 °C. Humidity ranges from 10% to 13%. (Omar et al., 2018). In the winter, the highest temperature ranges between 20 °C and 28 °C, and the lowest between 8 °C and 14 °C. The temperature in the winter occasionally goes down to as low as −2 °C, while the humidity ranges between 40% and 49% (Omar et al., 2018). Meanwhile, the climate of Al-Qassim region is characterized by a rainy winter, and a low-humidity summer (Imam et al., 2012).

Studies on the prevalence of *Eimeria* spp. in camels from other countries reported different infection rates. For example, the reported range of *Eimeria* infections from dromedary camels in Iran is 9.51%–63% (Khodakaram-Tafti et al., 2000; Yakhchali & Cheraghi, 2007; Borji et al., 2009; Yakhchali & Atari, 2010; Kheirandish, Nourollahi-Fard & Faryabi, 2012; Sazmand et al., 2012; Radfar & Gowhari, 2013). *E. cameli* was reported in 11% of examined camels in Uganda (Nakayima et al., 2017). Djerbouh et al. (2018) reported *E. dromedarii* and *E. cameli,* in 9.6% of the samples, and in Egypt, Abbas et al. (2019) reported *E. cameli*–like parasites in 31%, *E. rajasthani* in 18%, and *E. dromedarii* in 14% of examined animals. These different infection rates may reflect overall differences in geographical distribution and the influence of the variation in environmental conditions (humidity, temperature, oxygen level, and type of soil) affecting oocyst sporulation.

*Eimeria* spp*.* were distinguished by morphological characteristics. *E. cameli* had the largest oocysts, characterized by a dark brown, double thick wall and presence of micropyle. The other two *Eimeria* spp. were smaller; *E. rajasthani* was oval, with micropyle and polar cap, and *E. pellerdyi* was oval without micropyle and polar cap. The main limitation of this study lies in that the molecular analysis were not performed to supplement morphological analysis. Recently, molecular characterization has been widely used to ensure precise species classification, particularly where morphological differentiation is difficult due to similarities in shape and size (Ogedengbe, Hanner & Barta, 2011; Kokuzawa, Ichikawa-Seki & Itagaki, 2013). Further phylogenetic studies might shed light on the evolution and host specificity of *Eimeria* spp. in mammalian hosts.

## Conclusions

Our result gives an overview of camel eimeriosis in Riyadh and Al-Qassim, Saudi Arabia, but much more studies are needed to improve the understanding of the impact of eimeriosis on camel health, reproductive performance, meat and milk production, predisposition to other diseases and associated economic losses. Molecular based studies are recommended to elucidate the evolutionary traits in *Eimeria* spp.

##  Supplemental Information

10.7717/peerj.10347/supp-1 Supplemental Information 1 Raw data

## Additional Information and Declarations

### Data Availability

The following information was supplied regarding data availability: Raw data is available as a Supplemental File.
